# Understanding human – bat interactions in NSW, Australia: improving risk communication for prevention of Australian bat lyssavirus

**DOI:** 10.1186/1746-6148-10-144

**Published:** 2014-07-02

**Authors:** Emma K Quinn, Peter D Massey, Keren Cox-Witton, Beverley J Paterson, Keith Eastwood, David N Durrheim

**Affiliations:** 1 NSW Public Health Officer Training Program, NSW Ministry of Health, North Sydney, Australia; 2 School of Public Health and Community Medicine, University of New South Wales, North Sydney, Australia; 3 Population Health, Hunter New England Local Health District, North Sydney, NSW, Australia; 4 Wildlife Health Australia (formerly Australian Wildlife Health Network), Mosman, North Sydney, NSW, Australia; 5 Hunter Medical Research Institute, University of Newcastle, NSW, Australia; 6 Health Protection NSW, North Sydney, NSW, Australia

**Keywords:** Lyssavirus, Bats, Australia, Risk, Communication, Prevention

## Abstract

**Background:**

Australian bat lyssavirus (ABLV) infects a number of flying fox and insectivorous bats species in Australia. Human infection with ABLV is inevitably fatal unless prior vaccination and/or post-exposure treatment (PET) is given. Despite ongoing public health messaging about the risks associated with bat contact, surveillance data have revealed a four-fold increase in the number of people receiving PET for bat exposure in NSW between 2007 and 2011. Our study aimed to better understand these human – bat interactions in order to identify additional risk communication messages that could lower the risk of potential ABLV exposure. All people aged 18 years or over whom received PET for non-occupation related potential ABLV exposure in the Hunter New England Local Health District of Australia between July 2011 and July 2013 were considered eligible for the study. Eligible participants were invited to a telephone interview to explore the circumstances of their bat contact. Interviews were then transcribed and thematically analysed by two independent investigators.

**Results:**

Of 21 eligible participants that were able to be contacted, 16 consented and participated in a telephone interview. Participants reported bats as being widespread in their environment but reported a general lack of awareness about ABLV, particularly the risk of disease from bat scratches. Participants who attempted to ‘rescue’ bats did so because of a deep concern for the bat’s welfare. Participants reported a change in risk perception after the exposure event and provided suggestions for public health messages that could be used to raise awareness about ABLV.

**Conclusions:**

Reframing the current risk messages to account for the genuine concern of people for bat welfare may enhance the communication. The potential risk to the person and possible harm to the bat from an attempted ‘rescue’ should be promoted, along with contact details for animal rescue groups. The potential risk of ABLV from bat scratches merits greater emphasis.

## Background

Australia is home to over 70 bat species, ranging from large fruit-eating flying foxes, to small insect eating bats [1]. Some are listed as threatened species under State [2] and Commonwealth [3] legislation. Urban spread and habitat loss has affected migratory and foraging behaviour of flying foxes, leading to the establishment of camps in metropolitan areas near stable food sources [4], which can cause community concern. In Australia reports of flying foxes damaging orchards and getting caught in fruit tree netting are becoming increasingly common [5].

Australian bats are natural hosts for a range of zoonoses including Australian bat lyssavirus (ABLV) and Hendra virus [6,7]. Unlike Hendra virus, where transmission to humans has only occurred via infected horses, there have been three confirmed human cases of ABLV infection resulting from direct contact with bats. All were reported from Queensland and all subsequently died from the infection. Two of the cases were reported in the mid-1990s in females aged 30-40 years [8,9] and a child was infected by a bat in north Queensland in late 2012 and died in early 2013 [10].

ABLV is a member of the *Lyssavirus* genus of the *Rhabdoviridae* family that also includes the rabies virus. ABLV has been detected in flying foxes and small insectivorous bats in Australia [11,12]. Studies suggest <1% of wild-caught flying foxes are likely to carry the disease [11], but sick, injured or orphaned bats are much more likely to be infected [12,13]. Bats infected with ABLV may show a range of clinical signs including overt aggression, paralysis, paresis, seizures and tremors [12]. Human encounters are more likely to occur with bats that are sick (i.e. with ABLV infection, heat stress or other illness) due to the increased likelihood of sick animals being found on the ground [14-16].

ABLV is transmitted to humans through the saliva of the infected animal usually via a bite or scratch on the skin. Lyssavirus infection in humans almost always results in a fatal acute viral encephalomyelitis, unless post-exposure treatment (PET) is given [17]. In Australia it is recommended that PET with human rabies immunoglobulin and/or rabies vaccine is given to people who have been bitten or scratched by a bat, or where mucous membranes or broken skin have been contaminated with bat saliva [17,18]. For people at higher risk of exposure to ABLV from bats (e.g. wildlife carers, veterinarians, wildlife officers, bat ecologists), pre-exposure vaccination is recommended [18-20]. Bats should only be handled by vaccinated and trained people, however, even in this group, a bite or scratch warrants additional vaccine doses [18,21]. Recommended safety precautions include wearing protective gloves and clothing, and taking every effort to avoid being bitten or scratched.

Public health messages regarding the risk of bat contact are available from Australian State and Territory Government health and agriculture agencies [22-29], from non-government organisations [30] and from other sources [31]. These communication materials focus on human protection measures such as: education about lyssavirus transmission; advice on avoiding contact with bats; contacting a wildlife rescue group; and seeking medical advice. Examples of messages include ‘don’t touch or handle bats’, ‘if bitten or scratched take precautions by washing the wound with soap and water and apply antiseptic’ and ‘immediately seek medical care’. These communication messages rely on members of the public actively seeking more information regarding ABLV. However, in addition to this strategy, active media campaigns are often conducted during times of greater exposure risk, for example during the bush fire season and when trees are in fruit, or in response to an exposure event [32,33]. These media campaigns highlight the message ‘don’t touch or handle bats’.

Despite this advice, surveillance data reveal an ongoing exposure to potential ABLV infection in Australia [15,34-38]. The number of New South Wales (NSW) residents receiving PET for exposure to a bat in Australia, increased from 31 people in 2007 to 131 in 2011 with the majority of these exposures due to people rescuing bats tangled or trapped in fencing, barbed wire or netting [34]. A recent survey has indicated that 25% of people reporting previous exposure to bats said they would handle a bat again if it was trapped or injured [35].

By investigating human–bat interactions we aim to identify appropriate risk communication messages that could positively influence people’s behaviour towards reducing bat exposure and thus the potential risk of ABLV infection [39]. To date there have been no published qualitative studies in Australia examining human-bat interactions. Given the increasing number of bat exposures reported in NSW residents we sought to build on recent work [35] to further understand why human-bat exposures occur, in order to enhance public health risk communication strategies.

## Methods

### Interview sample frame and selection method

We purposively sampled all who received PET for potential ABLV exposure associated with bats in Hunter New England Local Health District for the period July 2011 to July 2013. Eligible participants were identified from the NSW Notifiable Conditions Incident Management System, using the sampling criteria above. People were excluded if they were under 18 years of age, had an overseas or occupational exposure, or could not be contacted after three attempts. From this sample frame, all eligible people were invited to participate in a telephone interview.

### Interview instrument and administration

A semi-structured interview was administered to participants and explored the following: (i) the circumstances that resulted in the individual coming into contact with a bat and the reasons why they touched or handled it; (ii) the appropriateness of current public health messaging regarding bats and ABLV; (iii) any new or different risk reduction strategies that could be used for potential ABLV exposure; and (iv) the most appropriate methods for disseminating public health information about bats and ABLV. Participants were interviewed during November and December 2013. Interviews were recorded and transcribed.

### Qualitative analysis of findings

Interview data were thematically analysed by two independent investigators. Investigators PM and EQ separately coded the data, using an open coding system whereby the codes were refined iteratively as the notes were re-analysed. Coding was then compared between investigators, deconstructed and reconstructed and once the coding system was finalised all notes were re-coded. Investigators then discussed the relationships or patterns between coded categories to enable aggregation of the data into overarching themes from the study and specific quotes were drawn from the transcribed data to illustrate these themes [40]. Interviews continued until data saturation was achieved. The themes reported below reflect both the number of participants reporting data and how strongly the participants reported the theme.

### Ethics

Ethical approval for this study was given by the Hunter New England Local Health District Human Research Ethics Committee (Reference no. LNR/13/HNE/356).

## Results

### Participation in the study

From the initial sample frame (n = 32), eleven people were excluded for reasons described above. Of the 21 remaining eligible participants, 16 consented to participate and were interviewed; further data were not collected on non-responders (n = 5). Interview duration ranged from 20 to 45 minutes. Participants ranged in age from 35-85 years and 10 were female.

### Exposure events

Notification data revealed twelve participants had initiated the contact with the bat and four reported accidental contact. Twelve participants had contact with a flying fox, nine of these exposure events were related to fruit tree netting entanglement. Four participants had contact with an insectivorous microbat, with all of these exposures related to household roosting. If available, the bat associated with a human exposure event will be tested for ABLV. In NSW, ABLV testing of bats is conducted by the NSW Department of Primary Industries (DPI) and the Commonwealth Scientific and Industrial Research Organisation - Australian Animal Health Laboratory. Results are communicated by the NSW DPI to NSW Health.

### Thematic analysis

Six themes emerged from the participant interviews and these are discussed below in detail. The type of bat contact (accidental or human initiated) influenced the participant’s experience and we provide comment for these two groups where appropriate.

#### Bats are considered common

Most participants reported bats being widespread in their environment and associated them with night time or when trees are in fruit. Some participants reported a ‘nuisance’ factor with large bat colonies *“raiding fruit trees”* and *“spew(*ing*)* [spitting/vomiting] *on the car”*.

#### Need to save the bat

Being *“an animal lover”* was a strong theme for the participants who initiated the contact with the bat. These participants expressed an overwhelming need to protect or save the bat from further distress, harm or pain, e.g. *“because I was worried about other wildlife attacking it see”*, and *“I just didn’t want to see it suffer lying there in the heat”*. Participants described continuing rescue attempts despite being repeatedly scratched, *“Oh well I just continued to try to get it out of the net”.* Participants often used the pronoun “he” or “she” when referring to the bat e.g. *“oh..I thought he doesn’t look comfortable”* and *“we didn’t want him to die like that you know”*. Participants justified their attempted ‘rescue’ by equating it with other animals *“I would have done the same if I had seen a wounded bird”*.

#### Bats can scratch and bite you!

Participants who had experienced an accidental bat contact reported the contact as *“unbelievable”* and that *“you don’t expect anything to happen to you, you know, like this”*. Participants who had initiated the bat contact reported a misguided “*confidence that I could handle* [the bat]*”* and myths about bat behaviour such as *“baby bats don’t bite”* that led them to feel confident in handling the bat. These participants were particularly surprised at the unpredictable nature of the contact *“lo and behold…it latched onto my thumb”,* and *“next moment its wing came out at full length and swiped me”*. The few participants who reported using some kind of personal protection (such as gardening gloves) were very surprised that the bat *“bit straight through the gloves”.*

#### Bat contact and disease risk

Most participants associated bats with *“some sort of nasty virus”* and *“knew enough that they were germy”*. However nearly all (13/16) participants had no specific awareness of ABLV prior to the exposure event. As one participant described, he *“knew about diseases,* [but] *I did not know about Lyssa”*. This overall lack of awareness and knowledge led some people to present to healthcare only because they were seeing a general practitioner for other reasons. Participants often associated bats with Hendra virus but not with ABLV e.g. *“the only thing I knew about bats was Hendra virus”*, and in some cases confused the two viruses e.g. “*oh hang on is that* [ABLV] *the thing to do with horses”*.

Six participants reported that hospital staff seemed unaware about the risk of ABLV from bat contact. As one participant explained, *“I don’t think she* [hospital staff member] *knew what to do to be honest at that stage”,* and another participant said that hospital staff *“need to know if someone comes to them and says I have been bitten by a bat…they should be more effective and more discerning”*.

Emerging from the findings for both groups of participants was a lack of concern about the association between bat scratches and risk of disease. Participants reported *“it didn’t really enter my brain I’m guessing because it was a little graze”* and *“I was pretty much unconcerned, because it wasn’t a deep gash or anything like that”.* Participants did not know that *“it was as simple as saliva and scratches”*.

#### Bat contact – fear, fault and next time

Many participants expressed a fear of bats prior to their exposure event and described them as *“a dangerous animal”,* and that some of this fear related to *“movies I have seen”*. This was most evident for participants who had accidental contact with the bat, where participants exclaimed *“I just wanted to get it out of here!”* and *“I was horrified”* [by the bat contact]. Both groups of participants expressed a fear that *“bats carried some kind of disease”* but *“didn’t realise it was that bad…a deadly virus!”* This led to a range of post-exposure actions immediately after the contact, where participants reported “*well I just washed off whatever*” to *“I was concerned enough to keep rubbing that antibacterial* [gel]”.

Participants who initiated the contact with the bat described it as their *“own stupid fault”* because they *“just scared it, I just freaked it* [the bat]”, while some participants blamed the bat for the contact: *“I was just trying to help but he just bit me”* and in some instances this led to retaliation against the bat. Participants did not strongly report pain associated with the bat bite or scratch, but *“that* [PET injections] *was more painful than the actual bite!”*

Both groups of participants expressed changes in their emotional state after the event occurred, as a result of the ‘life-threatening’ nature of the event. One participant expressed this ongoing worry as *“every time I get sick I think, I hope I haven’t got the Lyssavirus”*. For participants initiating the contact with the bat they explained a change in risk perception and that ‘next time’ they would do things differently, from *“don’t touch them, everything happens for a reason”* and *“[they’re] like snakes, leave them alone”* to an aggressive response of *“get a big stick and just kill them”*.

#### Communication strategies

Nearly all participants recalled the media associated with the Queensland case of the child who died in March 2013, without being prompted. For example; *“I remember seeing the picture on the TV about the poor young kid who got scratched by a bat”*. However most participants did not relate this specifically to ABLV infection, but acknowledged that this media *“was the only thing that made me aware of it* [i.e. risk of some disease associated with bats]*”*. In relation to the exposure event, participants strongly reported the need *“to get through to children to not make the same mistake we did”*, because *“young kids are always curious, that they don’t go and pick it up or touch it”* [the bat].

Overall, participants from both groups strongly believed that the level of awareness of ABLV needs to be increased, so that *“people out there….realise how serious it is”*. Some key messages suggested by participants were: (i) *“ring WIRES and just leave it there!”* (i.e. Wildlife Information Rescue and Education Service) and (ii) the *“importance of immediate medical assistance”* if bitten or scratched. A few participants thought it might be helpful to mention the *“relative inconvenience……and expense of the treatment”*.

*“Local radio”* or a *“local newspaper”* were reported as the most effective methods to communicate information to the public as they “*attract people because it’s personal stuff”* i.e. cover personal interest stories. At the time of the event, participants *“first…went to some websites”* and then *“did another internet search”* for more information on bats. Timing of public health communication was also seen as key by the participants, *“every time when it’s season….they* [bats] *come around”* [in reference to fruit season]*, “in spring and summer I suppose”.*

## Discussion

Participants in this study reported that bats are common in their environment and that there is some fear related to bats, although scratches were not seen as posing a disease risk. The tendency to empathise with an animal’s suffering and be concerned for its welfare appeared to be the strongest factor underlying participants’ attempts to save bats. Existing risk communication materials may need to be re-framed to appeal to the affective/emotional side of individuals who feel compelled to ‘rescue’ bats.

Individuals understand risks according to the nature of the risk itself and a combination of cultural and social factors [41]. The dimensions to risk perception consist of knowledge; the probability of the risk and whether it is observable; sense of dread or fear from the risk; whether effects are immediate or delayed; and whether there are fatal consequences [42]. In our study, bats were considered prevalent in the environment and participants reported fear of them and the *“diseases they carry”*, but most reported no specific awareness of ABLV. These findings are supported by a recent Queensland study published by Young et.al [43], which found that of those previously unexposed Queensland adults participating in a telephone survey (n = 700), over half reported bats as a risk to human health. However only 10% specifically reported ABLV as a risk and only 47% of participants recalled ABLV when prompted. Participants in our study also reported a lack of association between bat scratches (as opposed to bites) and disease risk, with most participants dismissive of this type of injury. Despite all participants receiving PET, some reported ongoing worry related to the potential fatal consequences of ABLV infection.

Participants initiating the ‘rescue’ of the bat reported a deep concern for the bat’s welfare, wanting to protect the bat from further harm or distress. Empathy for animal welfare is common among humans and increasing levels of empathy have been shown to be associated with animals that have phylogenetic relatedness to humans [44]. This may partly explain why some participants felt a duty to protect the welfare of the bat and used animate pronouns to refer to the bat when describing the ‘rescue’ e.g. *“hedoesn’t look comfortable”.*

It is important to understand the societal response to the risk of zoonoses, including media coverage and government education campaigns, as these factors can influence an individual’s perception regarding risk [45,46]. Participants in this study were aware of Hendra virus and its association with bats, but not specifically of ABLV. This may not be surprising given the increased media coverage during the Hendra outbreaks in 2011 [47]. The *“poor little boy”* who died in Queensland was recalled by participants when they were asked to suggest educational strategies for raising awareness of ABLV. The study published by Young et al. [43] also reported that only 10% of survey participants reported ABLV as a potential risk from bat exposure compared to 30% of participants reporting Hendra as an exposure risk.

Participants emphasised the need to *“educate and protect kids”,* in contrast to surveillance data that indicates that more than 90% of reported exposures in NSW occur in adults [34]. However one of the three ABLV-associated deaths involved a child [10], where the young boy did not report the exposure event (i.e. bat scratch) early enough to be given PET for ABLV. There is also a strong cultural value around protecting and supporting the health of more vulnerable individuals or groups (particularly children) in the population [48] which may partly explain the expressed need to educate children about ABLV. These findings are also supported by the recent published study from Young et al. [43], whereby the majority of participants in their study (624/700) reported that they would handle a bat to protect their family, children or pets from harm.

Participants that had initiated contact with the bat reported substantially different actions they would take ‘next time’ if they encountered a bat in need of help e.g. *“don’t touch them”* and *“let the professionals come and do it”*. This change in risk perception due to a life threatening event can be explained by the ‘health belief model’ [49] and ‘protection motivation theory’ [50]. These theories describe how risk perception and behaviour changes when: 1) a threat is considered severe, 2) there is a possibility that it may occur, and 3) there is an action that the individual can take in the future to mitigate that risk. This is especially important given that the participants who initiated contact with the bat reported concern about the unpredictable nature of the contact (i.e. bats with a large wing span or very sharp teeth that can bite or scratch through or around inadequate personal protective equipment).

Current educational messages promoted by government departments across Australia [22-25,51] are based on rational thinking and notions of self-protection when encountering bats and may be effective for most of the population, including ‘don’t touch bats’ , ‘if bitten or scratched wash the wound’ and ‘seek medical care’. However most of the participants in this study who had experienced bat bites or scratches requiring treatment, reported a deep concern for the bat’s welfare. Therefore government departments in public health and animal health may wish to consider reframing existing messages to provide a balance between human protection measures and bat welfare [52]. This may help further deter people from ‘rescuing’ bats, for example “you will hurt the bat and place yourself at risk of ABLV if you attempt to rescue the animal”.

The results from this study also suggest that further emphasis on the potential risk of ABLV transmission from bat scratches may be helpful. However, it is important to balance the warning message about ABLV against messages of self-protection [53] so that the risk is not amplified in the community to a destructive level potentially endangering the lives of bats [54]. Continuing to link the risk education message with an alternative action is also important (i.e. providing the contact details of wildlife rescue groups), both from an animal welfare perspective and also in providing a feasible alternative for people who want to help an injured bat. Proactive and responsive local media i.e. “push communication strategies” during times of high exposure risk, such as bush fires, fruit tree season and bat breeding season, may help maintain levels of awareness about ABLV and knowledge of appropriate action when a bat is encountered or an exposure has occurred [52].

### Limitations

This study was conducted in northern NSW and with a relatively small number of participants. However the Hunter New England Local Health District is the most populous regional health authority in NSW and the two-year sample timeframe ensured maximum recruitment of eligible participants, while providing the best opportunity for accurate recall of exposure events. The purposive sampling method is not without potential bias; only those whose exposure had been notified to the local public health unit were included and no data were collected on non-responders. It is likely that un-notified bat exposures occur in regional NSW; however identification of these people for recruitment into this study would be very difficult. The findings may not be generalizable across all geographical regions or contexts. However we anticipate that our results could be used with some validity by public health practitioners and policy makers in NSW and possibly other States and Territories in Australia to enhance risk communication regarding bats and ABLV exposure risk.

## Conclusions

The tendency of people to empathise with an animal’s suffering appears to be the strongest factor explaining why people in this cohort engaged in ‘rescuing or saving’ bats. This issue needs to be taken into account in the development of risk communication messages. The contact details for animal rescue groups should continue to be promoted in all ABLV risk communication materials as these people are vaccinated and have the expertise to handle wild animals. Risk communication messages also need to further increase the awareness about the potential risk of ABLV from bat scratches.

## Competing interests

BP was supported by a Hunter Medical Research Institute research fellowship.

## Author’s contributions

EQ was the lead investigator for the research study. EQ was involved in conducting all interviews and the thematic analysis, drafting the manuscript and finalising it with all authors for submission. PDM is a co-investigator for the research study. PDM was involved in the design of the study, thematic analysis of all interviews, editing and finalisation of the manuscript for submission. KCW is a co-investigator for the research study. KCW was involved in the design of the study, editing and finalisation of the manuscript for submission. BP is a co-investigator for the research study. BP was involved in the design of the study, editing and finalisation of the manuscript for submission. KE is a co-investigator for the research study. KE was involved in the design of the study, editing and finalisation of the manuscript for submission. DD is a co-investigator for the research study. DD was involved in the design of the study, editing and finalisation of the manuscript for submission. All authors read and approved the final manuscript.
